# Psychotropic medication in the French child and adolescent population: prevalence estimation from health insurance data and national self-report survey data

**DOI:** 10.1186/1471-244X-9-72

**Published:** 2009-11-17

**Authors:** Eric Acquaviva, Stéphane Legleye, Guy R Auleley, Jean Deligne, Didier Carel, Bruno Falissard B

**Affiliations:** 1 Inserm U669, PSIGIAM, Paris, France; 2 Université Paris-Sud, UMR-S0669, Paris, France; 3 Hôpital Robert Debré, Service de psychiatrie, Paris, France; 4 Observatoire Français des Drogues et toxicomanies, Saint Denis, France; 5 Caisse Nationale du RSI, La Plaine-Saint Denis, France; 6 Régime Social des Indépendants Nord - Pas de Calais, Lille, France; 7 Urcam de Franche-Comté, Besançon, France; 8 Hôpital Paul Brousse, Département de santé publique, Villejuif, France

## Abstract

**Background:**

The aim of this work is to estimate the French frequencies of dispensed psychotropic prescriptions in children and adolescents. Prevalence estimations of dispensed prescriptions are compared to the frequencies of use of psychotropic reported by 17 year-old adolescents.

**Methods:**

Prescription data is derived from national health insurance databases. Frequencies of dispensed prescriptions are extrapolated to estimate a range for the 2004 national rates. Self-report data is derived from the 2003 and 2005 ESCAPAD study, an epidemiological study based on a questionnaire focused on health and drug consumption.

**Results:**

The prevalence estimation shows that the prevalence of prescription of a psychotropic medication to young persons between 3 and 18 years is about 2.2%.

In 2005, the self-report study (ESCAPAD) shows that 14.9% of 17 year-old adolescents took medication for "nerves" or "to sleep" during the previous 12 months. The same study in 2003 also shows that 62.3% of adolescents aged 17 and 18 reporting psychotropic use, took the medication for anxiety and 56.8% to sleep. Only 49.7% of these medications are suggested by a doctor.

**Conclusion:**

This study underlines a similar range of prevalence of psychotropic prescriptions in France to that observed in other European countries. Nevertheless, the proportion of antipsychotics and benzodiazepines seems to be higher, whereas the proportion of methylphenidate is lower.

Secondly, a disparity between the prevalence of dispensed prescriptions and the self-report of actual use of psychotropics has been highlighted by the ESCAPAD study which shows that these treatments are widely used as "self-medication".

## Background

Psychotropic medication in children and adolescents is a matter of concern in most Western countries. However, few randomized controlled trials have evaluated pharmacological treatments in children and adolescents for psychiatric disorders [1,2]. In addition, the official indications of most psychotropic drugs for children are not clear and off-label prescriptions have expanded fast during the last decade [3-5]. Recently, these problems have been pinpointed as particularly important for some drugs, since different countries have issued warnings on serotonin reuptake inhibitor antidepressants [6-8].

In France, little data concerning the frequency of psychotropic treatments in children and adolescents is available. In 2002, a study [9] based on medication reimbursement claims registered in the database of a regional branch of the main health care insurance reported widespread use of psychotropic medication by French adolescents. In addition, several epidemiological studies have raised concerns about psychotropic consumption in France: legal drug use in France is among the highest in the world and it was the highest in the European Union in 1997 [10-12].

Unfortunately, no national study on psychotropic prescriptions has as yet been conducted in children and adolescents in France. The aim of this work is to estimate the national frequencies of prescription of psychotropic treatment (antidepressants, stimulants, antipsychotics and benzodiazepines) in children and adolescents. Data is derived from several national health insurance databases. Results are compared to responses given by adolescents to a large national epidemiological study.

## Methods

### Study settings and design

In France, the national health insurance system comprises three main agencies: CNAM-TS *(Caisse Nationale d'Assurance Maladie) *for salaried employees, RSI *(Régime Social des indépendants) *for independent and self-employed workers and MSA *(Mutuelle Sociale Agricole) *for self-employed and salaried workers in the agricultural sector. These three agencies account for 96.6% of the French population (86.2% for CNAM TS, 4.4% for RSI, 6.0% for MSA) [13].

Children and adolescents under 18 are affiliated to their parent's insurance funds. All prescriptions issued by a physician and dispensed by pharmacies are coded and put into a database that is specific to each agency. In each insurance fund, each claim is specifically coded, registered in a computerized database and linked to beneficiary identity.

In this study, we performed two cross sectional studies analyzing stimulant, benzodiazepine, anti-psychotic and antidepressant prescriptions in children and adolescents under 19: the first was conducted among affiliates of the RSI in 2004, and the second among affiliates of the CNAM-TS in the "Franche Comté" region in 2005.

As a second step, data derived from the 2003 and 2005 ESCAPAD study, an epidemiological study based on a self-completed questionnaire focused on health and consumption of medication, was analysed. This survey is performed regularly (2000, 2001, 2002, 2003 and 2005) on a representative sample of French adolescents during a "civil service" day for all French subjects aged 17 and 18. This day replaces the former mandatory military service check-up, and concerns both boys and girls. The 2003 and 2005 surveys were used, including respectively samples of 15 710 and 29 393 individuals [14,15].

The present study focuses on five particular items in the questionnaire:

- Do you usually take medication for a psychological problem (i.e. at least once per week over the last six months)?

- Have you taken drugs for nerves or to sleep in the last 12 months? If you have, how many times? once or twice, between 3 and 5 times, between 6 and 9 times, 10 times and more.

- Have you taken drugs for nerves or to sleep in the last 30 days? If you have, how many times? once or twice, between 3 and 5 times, between 6 and 9 times, between 10 and 19 times, between 20 and 29 times, every day.

- Who gave you the last medication you took? A physician, one of your parents, one of your friends, nobody (I took it by myself), other situation ?

(This question was asked only in 2003)

For what reason did you take this medication? (several answers possible): to cure some illness, against stress, to sleep, to get some stimulation, for a party, because of a traumatic event, other reason)?

(This question was asked only in 2003)

### Data management

Concerning data from the health insurance providers, the prescriptions were collected using ATC classification. All prescriptions during a given year with an ATC code beginning with N05A (antipsychotics), N05B (anxiolytics), N05C (hypnotics), N06A (antidepressants), N06B (psychostimulants) were included. This classification was refined and medications were finally categorized in the following groups: methylphenidate, serotonin specific reuptake inhibitors (SSRI), benzodiazepines and anti-psychotics. It should be noted that methylphenidate is the only psychostimulant authorized for children in France.

### Statistical methods

A model was designed to estimate frequencies of prescriptions according to age and gender: a "year effect" was calculated for each class of medication. The "year effect" is the ratio between the 2004 and 2005 "Franche Comté" RSI prevalence of dispensed prescriptions.

As a second step the ratios of prescriptions according class of medication were calculated between RSI in the "Franche Comté" region in 2004 and RSI in France in 2004. These ratios were used to estimate a "geographical effect" for each class of medication.

Complete data concerning the prescription of a medication according to class, subject age (in years) and gender for RSI in 2004 was obtained. Combining these data with the "year effect" and the "geographical effect", the levels of consumption according to class of drug, age (in years) and gender for the CNAM-TS in 2004 was then estimated.

The mathematical model is as follows:

P = frequency of prescription

i0 = Franche - Comté; i1 = France

j0 = 2004; j1 = 2005

We have to calculate the estimation of Pcnam (i1, j0)

Year effect = Prsi (i0, j0)/Prsi (i0, j1)

Geographical effect = Prsi(i1, j0)/Prsi(i0, j0)

Pcnam (i1, j0) = Pcnam (i0, j1) × year effect × geographical effect

Or

Pcnam (i1, j0) = Pcnam (i0, j1) × Prsi (i1, j0)/Prsi (i0, j1)

We checked that thedistribution between France and Franche Comté in terms of age and activity (Table 1) was similar in order to extrapolate prevalence of prescriptions from Franche Comté to France [16].

**Table 1:** Distribution of age and activity in France and Franche Comté

	Franche Comté (% of the population)	France (% of the population)
**Age**		
0-19	25.2	25.1
20-39	25.9	26.5
40-59	27.9	27.8
>60	21	20.7

**Working population**	47.5	52.5
- Employed	42.6	42.1
- Unemployed	4.9	5.4
**Non-working population**	52.5	52.5
- Retired	20.8	
- Students	7.7	20
- Others	20.8	8.2

**Psychotropic prevalence**	2.1	2.5
Age: 0-18		

We also compared the overall prescriptions of psychotropics between France and Franche Comté in the 2004 RSI database for children and adolescents (Table 1).

Concerning the MSA insurance system, it was hypothesised that the frequencies of psychotropic prescriptions fell between the RSI frequencies and the CNAM-TS estimations.

The range of the overall prevalence of psychotropics in France was estimated using the lowest and the highest hypothesis of the prevalence of prescriptions in the MSA.

Finally, frequencies of prescriptions according to psychotropic class, age and gender were combined for these three insurance systems using a weighted mean (according to their relative importance in terms of numbers of insured persons). Since the MSA, CNAM-TS and RSI cover about 96.6% of the French population, this should provide fairly acceptable estimates of the level of prescriptions in France.

## Results

### Description of the population of the health care insurance sample

In France in 2004, 536 606 children and adolescents aged between 0 and 18 were affiliated to the RSI, 13 533 415 to the CNAM-TS (259 885 to the CNAM-TS Franche-Comté) and 944 075 to the MSA [15].

There were 15, 124 052 children and adolescents aged from 0 to 18 in France in 2004 [16].

Hence our model accounts for about 99,3% of the French population of this age.

### All psychotropics (table 2)

**Table 2:** Estimation of the French prevalence of psychotropic use in 2004

age	Boys (‰)	Girls (‰)	All (‰)
**0-4**	[6.4 ; 6.8]	[6.2 ; 6.6]	[6.3 ; 6.7]
**5-9**	[14.4 ; 15.3]	[12.2 ; 13.0]	[13.3 ; 14.2]
**10-14**	[18.6 ; 19.9]	[20.1 ; 21.5]	[19.3 ; 20.6]
**15-18**	[25.7 ; 27.4]	[49.3 ; 52.6]	[37.8 ; 40.3]
**all**	**[18.5 ; 19.7]**	**[24.6 ; 26.2]**	**[21.5 ; 22.9]**

The overall annual prevalence of prescriptions of psychotropic medication to young persons between 0 and 18 years is about 2.2%. This annual rate of prescription rises from approximately 1.2% to 5% between 5 and 18 years. From 0 to 12 years, the rate of prescription is higher in boys. At 13, the rate becomes higher in girls. At 18 the gender ratio is about 2:1.

### Methylphenidate (table 3)

**Table 3:** Estimation of the French prevalence of methylphenidate use in 2004

age	Boys (‰)	Girls (‰)	All (‰)
**0-4**	[0.054 ; 0.055]	[0.027 ; 0.028]	[0.040 ; 0.041]
**5-9**	[3.0; 3.1]	[5.1 ; 5.2]	[1.8 ; 1.9]
**10-14**	[3.7 ; 3.8]	[0.69 ; 0.71]	[2.7 ; 2.8]
**15-18**	[1.7 ; 1.8]	[0.27 ; 0.28]	[1.0 ; 1.1]
**all**	**[2.5 ; 2.6]**	**[0.38 ; 0.39]**	**[1.4 ; 1.5]**

The maximum prevalence of prescription of methylphenidate is obtained in boys aged 8 years, where it is about 0.65%. It can be noted that the age of the maximum prevalence of methylphenidate prescription is older in girls. The rate of prescription is always lower for girls. Most of the prescriptions are issued for individuals between the ages of 7 and 16.

### Benzodiazepines (table 4)

**Table 4:** Estimation of the French prevalence of benzodiazepine use in 2004

age	Boys (‰)	Girls (‰)	All (‰)
**0-4**	[0.070 ; 0.0071]	[0.075 ; 0.076]	[0.73 ; 0.74]
**5-9**	[0.47 ; 0.48]	[0.50 ; 0.51]	[0.48 ; 0.49]
**10-14**	[1.8 ; 1.9]	[1.8 ; 1.9]	[1.8 ; 1.9]
**15-18**	[5.7 ; 5.8]	[11.9 ; 12.2]	[8.9 ; 9.1]
**all**	**[2.1 ; 2.2]**	**[3.9 ; 4.0]**	**[3.0 ; 3.1]**

The rate of benzodiazepine prescription increases with age. The frequency of prescription is higher in boys until the age of 13. From 14 to 18 the rate is higher in girls. The gender ratio is about 2:1 at the ages of 17 and 18. At the age of 18, the frequency of prescription reaches approximately 1.2%.

### SSRI (table 5)

**Table 5:** Estimation of the French prevalence of SSRI use in 2004

age	Boys (‰)	Girls (‰)	All (‰)
**0-4**	[1.1 ; 1.2]	[1.2 ; 1.3]	[1.2 ; 1.3]
**5-9**	[2.2 ; 2.3]	[1.9 ; 2.0]	[2.1 ; 2.2]
**10-14**	[3.0 ; 3.1]	[3.5 ; 3.6]	[3.3 ; 3.4]
**15-18**	[8.2 ; 8.4]	[15.6; 16.0]	[12.0 ; 12.3]
**all**	**[4.2 ; 4.4]**	**[6.3 ; 6.5]**	**[5.2 ; 5.4]**

The prevalence of SSRI prescription increases with age. From ages 3 to 13 the frequency of prescription fluctuates between 0.15% to 0.25% and the rates are similar in boys and girls. From 14, the rate rises from approximately 0.4% to 1.4%. The percentage of prescriptions of SSRI reaches 2.2% in young women at 18, which is twice as high as in young men.

### Antipsychotics (table 6)

**Table 6:** Estimation of the French prevalence of antipsychotic use in 2004

	Boys (‰)	Girls (‰)	All (‰)
**0-4**	[0.62 ; 0.64]	[0.38 ; 0.39]	[0.51 ; 0.52]
**5-9**	[3.0; 3.1]	[1.2; 1.3]	[2.1 ; 2.2]
**10-14**	[4.5 ; 4.6]	[1.9 ; 2.0]	[3.2 ; 3.3]
**15-18**	[7.4 ; 7.6]	[5.9 ; 6.0]	[6.6 ; 6.8]
**all**	**[4.1 ; 4.2]**	**[2.5 ; 2.6]**	**[3.3 ; 3.4]**

The prevalence of the prescription of antipsychotics also increases with age. The frequency of prescriptions is always higher in boys than in girls.

### Self-report data collected by ESCAPAD

In 2005, 14.9% of the adolescents aged 17 years old reported having taken some form of medication for "nerves" or to sleep in the preceding 12 months (22.0% for girls, 8.0 for boys, p < 0.0001), 7.7% had taken this medication in the preceding 30 days (11.8% for girls vs 3.7% for boys, p < 0.0001) and only 2.2% had taken this medication at least 10 times in the previous 30 days (3.4% vs 1.1, p < 0.0001). A significant proportion of this medication (38%) involved plants or homeopathy.

In addition, only 2.7% of the adolescents reported that they were regularly taking some form of psychotropic medication (for at least 6 months) for psychological purposes; 3.0% reported that they were being followed by a physician for a psychological problem at the time of the survey.

### Reasons for the use of psychotropics (table 7)

**Table 7:** Purpose of the most recent psychotropic use among psychotropic users -- ESCAPAD 2003

	To cure an illness	Against anxiety	To sleep	To be stimulated	For fun	After a traumatic event	Other
**girls**	21.2%	71.5%	57.7%	8.5%	1.9%	0.9%	1.4%
**boys**	28.2%	40.1%	54.5%	9.4%	5.5%	0.4%	1.0%
**All**	23.3%	62.3%	56.8%	8.8%	3.0%	0.8%	1.3%

Results from the 2003 ESCAPAD study show that most of the psychotropic use that is drugs "for nerves or to sleep" reported by adolescents aged 17 and 18 years old was for anxiety or to sleep (it should be noted that the purpose of use was reported only for the most recent use) while about 10% of use was for fun or stimulation.

### Persons who suggested the most recent use of psychotropics (table 8)

**Table 8:** Persons who suggested the most recent use of psychotropics among psychotropic users between ages 17 and 18 -- ESCAPAD 2003

	Doctor	Relative	Friend	Own decision	Other situation
**girls**	51.1%	27.6%	2.9%	17.2%	1.3%
**boys**	46.3%	30.3%	3.8%	18.2%	1.4%
**all**	49.7%	28.4%	3.1%	17.5%	1.3%

About half of the psychotropics used by young people at 17 years are taken without a decision by a doctor. This proportion is negatively correlated with frequency of use: only 25.0% of adolescents who take some form of psychotropic medication almost daily are concerned.

## Discussion

To our knowledge, this is the first national estimation of psychotropic prescriptions in children and adolescents in France. Its originality is the comparison of official national health insurance data with epidemiological data based on a self-completed questionnaire. These epidemiological statistics provide some interesting information about the reasons for psychotropic drug use among adolescents.

The main limitation of the study is the restricted access to the CNAM-TS database (only one region) and the absence of access to the MSA database. However, we constructed a model which is liable to deal with this limitation: a range estimate is provided, which takes into account the absence of data from the MSA insurance

This calculation of a nationwide estimate highlights several trends:

In comparison with other European and North American countries, the frequency of overall psychotropic prescription in France is similar to that in United Kingdom (approximately 2%), Netherlands (2.9%), and Germany (2.0%). It is higher than Italy (0.3%) and lower than the USA (6.7%) [17-27].

A recent study conducted in France in MGEN affiliates found similar rates of overall psychotropic frequencies of 2.1% vs 2.2% in our study [28]. The frequency of methylphenidate and SSRI prescription is a bit higher in our study (0,1% for psychostimulants and 0.4% for antidepressants in the MGEN study versus 0.15% for methylphenidate and 0.5% for SSRI). For anxiolytics, the comparison is difficult because the MGEN study takes herbal medicines into account.

In France, the profile of prescriptions is however different. Antipsychotics and benzodiazepines seem to be prescribed at a higher level than in many European countries, whereas methylphenidate appears to be less prescribed. A hypothesis can be proposed to explain this point: under-diagnosis of ADHD and the use of symptomatic treatments such as antipsychotics and benzodiazepines to deal with externalized disorders in children and adolescents.

For methylphenidate prescriptions, the maximum of prevalence rate is obtained at 8 years for boys and at 10 years for girls. This probably reflects the delay in the diagnosis of ADHD between boys and girls.

The ESCAPAD survey is based on a self-completed questionnaire. Its analysis provides some additional and complementary information. Almost 15% of the adolescents aged 17 in 2005 took medication "for nerves or to sleep".

If homeopathy and plants are removed, we obtain prevalence for consumption of psychotropics of 9% in 17 year-old adolescents. This percentage is higher than the prescription prevalence derived from the social security databases (4.1-4.4% at the age of 17).

This suggests that either some adolescents obtain psychotropic drugs without medical prescriptions, or that they take psychotropic drugs legally obtained with earlier medical prescriptions.

Indeed, in 2003, 49.7% of the adolescents aged 17 who took some kind of medication for their nerves or to sleep in previous 12 months reported that they obtained it via a doctor the last time they took it (table 7); this corresponds to almost 4.5% of the 17 year-old adolescent population. This prevalence appears very close to the prevalence noted in the prescriptions (4.1%).

ESCAPAD also gave some indications about the purpose of psychotropic medication for adolescents. The main purpose is different between boys and girls. Girls use psychotropics to deal with anxiety whereas boys use pyschotropics to treat sleep disorders. The use of psychotropics for stimulation or for fun reaches 10% for girls and approximately 15% for boys. It would be interesting to study how this type of use evolves, and which drugs are the most widely consumed in these indications.

In addition, psychotropics can be used to treat physical heath problems in children.

We checked all official physical indications for the psychotropics studied and looked for information on non official indications.

It appears that tricyclic antidepressants are used to treat enuresis in children and could be marginally use to treat pain. In this study, only SSRIs were taken into account to avoid this bias. There are no physical indications found for this therapeutic class.

Methylphenidate has another official indication which is narcolepsy. We cannot know the proportion of methylphenidate used to treat narcolepsy. Empirically, it is likely to be very rare.

Antipsychotics have no official indication to treat physical health problems in children. Haloperidol is used although infrequently as an antiemetic drug.

Concerning benzodiazepines, the question is more difficult. Indeed, benzodiazepines are also indicated to treat epilepsy in children and in rare instances used as analgesic. In our study, it is impossible to know whether benzodiazepines are used to treat anxiety or epilepsy. To our knowledge, in France, there is no publication giving an estimation of the rates of benzodiazepines used against anxiety or epilepsy. Empirically, the proportion of benzodiazepines used to treat epilepsy is likely to be very low compared to psychiatric indications. In addition, other prevalence studies in children do not specify the aim of the prescription for benzodiazepines. Thus, the prevalence estimation in our study can be compared to other studies.

## Conclusion

As a conclusion, this study evidences a similar range of prevalence of psychotropic prescriptions to young people in France to that observed in certain European countries, a higher proportion of antipsychotic and benzodiazepine use in children and adolescents, and a lower proportion of methylphenidate use. It suggests an under-diagnosis of ADHD in France, and probably different approaches to treating certain psychiatric disorders in children and adolescents.

Secondly, a disparity between dispensed prescriptions and the self-report of actual use of psychotropics has been highlighted via data from the ESCAPAD study, showing that these treatments are widely used as "self-medication".

## Competing interests

The authors declare that they have no competing interests.

## Authors' contributions

SL, GA, JD, CD have made contributions for acquisition of data and analysis of data. EA, BF have made contributions for analysis and interpretation of data. EA, BF have been involved in drafting the manuscript and revising it critically for important intellectual content and have given final approval of the version to be published

## Pre-publication history

The pre-publication history for this paper can be accessed here:

http://www.biomedcentral.com/1471-244X/9/72/prepub
